# Vojta Therapy in Neuromotor Development of Pediatrics Patients with Periventricular Leukomalacia: Case Series

**DOI:** 10.3390/medicina57111149

**Published:** 2021-10-23

**Authors:** Elena De-La-Barrera-Aranda, Juan Jose Gonzalez-Gerez, Manuel Saavedra-Hernandez, Laura Fernandez-Bueno, Cleofas Rodriguez-Blanco, Carlos Bernal-Utrera

**Affiliations:** 1 Department of Morphological and Socio-Health Sciences, University of Cordoba, 14014 Cordoba, Spain; fisioelenacordoba@gmail.com; 2 Fisiosur I+D Research Institute, 04630 Almeria, Spain; juanjo@fisiosurid.es (J.J.G.-G.); clinicasaavedra@yahoo.es (M.S.-H.); 3 Department of Nursing, Physiotherapy and Medicine, University of Almeria, 04120 Almería, Spain; 4 Virgen del Rocío University Hospital, 41013 Sevilla, Spain; laurafernandez0895@gmail.com; 5 Deparment of Physiotherapy, Faculty of Nursing, Physiotherapy and Podiatry, University of Seville, 41004 Sevilla, Spain

**Keywords:** pediatric neurology, physical therapy, non-invasive therapy, vojta therapy, postural control

## Abstract

*Background and Objectives*: Vojta therapy is used by physiotherapists and is based on stimulation through peripheral pressure that leads to the activation of involuntary motor response patterns, thus triggering patterns of reflex locomotion, hence also called reflex locomotion therapy. Objective: To analyze the changes produced by Vojta therapy in the evolution of infant motor development in patients with maturational delay due to periventricular leukomalacia. *Materials and methods*: One session of Vojta Therapy per week for eleven months, patients’ neuromotor development was evaluated through the Denver II Test and the Baleys Scale. *Results*: A clinically significant increase in the development of the patients is observed. *Conclusions*: Neuromotor development seems to generate an adequate progression in the motor area.

## 1. Introduction

Periventricular leukomalacia is a necrosis of the white matter, located dorsal and lateral to the outer corners of the lateral ventricles [1]. Preterm birth and low birth weight are the main risk factors for triggering cerebral hypoxia, which is involved in establishing the lesion [2]. Prevalence in children under 28 weeks was 39.6%: 27.4% in children under 32 weeks, and 7.3% in children under 37 weeks. Other risk factors include gestational age, intrauterine infection, premature rupture of membranes, and chorioamnionitis [3]. Leukomalacia produces foci of infarction at the neurological level, where intracranial hemorrhage is one of the complications, and the sequelae that can be seen are descending degeneration of the corticospinal tract [4], the residual neurological symptomatology in the vast majority of patients is seen in the form of infantile cerebral palsy, psychomotor retardation, and visual disturbances. The degree of neurological damage and psychomotor retardation has been correlated with the severity of periventricular leukomalacia lesions [5].

Neurological damage is one of the main areas of activity of pediatric physiotherapy, within this area, Vodja therapy is one of the most popular methods of physiotherapeutic approach. Vojta therapy is based on stimulation through peripheral pressure that leads to the activation of involuntary motor response patterns, thus triggering reflex locomotion patterns, hence also called reflex locomotion therapy [6]. At the neurophysiological level different articles reflect how stimulation according to Vojta therapy induces sensorimotor changes, which have been verified after functional magnetic resonance imaging. The excitation of the stimulation point in the heel causes an increase in task-related activation of the pontomedullary reticular formation [7,8]. Measurement of the spinal transmission pathways of muscle responses after stimulation according to the Vojta method has led to the conclusion that one of the factors responsible for these responses is neural cross-transmission via the neurons of the long propriospinal tract [9].

Vojta therapy is used in physiotherapy to treat different pathologies. We found evidence on the treatment of subluxation of the hip joint, performing the first phase of reflex turning and reflex reptation, where centralization of the femoral head and a favorable influence on the formation of the acetabulum of the hip joint was obtained [10].

Another study talks about the impact of generating central patterns through stimulation of the pectoral area using the Vojta method, where the regularity and rhythmicity of nutritive and non-nutritive sucking are improved [11]. Other study shows how patients with Sotos syndrome with developmental delay who have been treated with the Vojta method improve the quality of motor patterns and cognitive activation [12]. Vojta therapy has also been used to improve the sitting position and diaphragm movement during inspiration in children with spastic cerebral palsy and is an effective treatment [13].

There is scientific evidence on Vojta therapy as a sensorimotor stimulation increasing reflex locomotion patterns. Still, there has been no research on how it explicitly influences motor development’s evolution, especially in pathologies that cause neurological damage, such as leukomalacia. 

We present a case series, concerning clinical motor and neurocognitive manifestations diagnosed with periventricular leukomalacia, where the importance of its evolution depends on interdisciplinary teamwork, early diagnosis, and early intervention. Given the clinical experience in the sector and the sequelae of patients diagnosed with periventricular leukomalacia, it is interesting to scientifically prove whether Vojta therapy might cause a favorable evolution in the motor development of infantile patients diagnosed with leukomalacia.

## 2. Materials and Methods

### 2.1. Study Design

Intervention case report series. The study was approved by the Andalucía Government (Spain) ethics committee (approve date on 15 October 2019; Coded CE2019/4127).

### 2.2. Objective

To analyze the changes produced by Vojta therapy in the evolution of infant motor development in patients with maturational delay due to periventricular leukomalacia.

### 2.3. Study Population

Pediatric patients were attending private physiotherapy consultations, diagnosed with a medical diagnosis of periventricular leukomalacia.

### 2.4. Selection Criteria. Inclusion and Exclusion

Inclusion criteria:

- A medical diagnosis of periventricular leukomalacia.
- Age: 0 months–4 years.
- Maturational delay.

Exclusion criteria:

- >4 years old.
- Other associated pathologies are not related to periventricular leukomalacia.
- No maturational delay.

### 2.5. Description of Patients

Male patients who come to the clinic with a medical diagnosis of leukomalacia and who, after assessment, show delayed motor development. A summary of initial characteristics can be check in Table 1.

- PATIENT 1: preterm male patient, age 21 months, corrected age 19 months. Birth weight 1398 kg. He came for consultation at ten months of age, corrected age seven months. He was diagnosed with periventricular leukomalacia. Diagnostic tests: cranial MRI (asymmetric ventricular system with dilatation of both lateral ventricles, more on the right, due to atrophy of the periventricular white matter about leukomalacia). Hyperintense areas in T1, hypointense in T2, and a signal drop in gradient echo sequences related to old hemorrhagic foci. Atrophy of the corpus callosum. All related to hypoxic-ischemic encephalopathy of prematurity.
- PATIENT 2: preterm male patient, 49 months, corrected age 36 months. He came for consultation at 36 months of age, corrected age to 33 months. Birth weight was 1750 kg. He was diagnosed with periventricular leukomalacia. On cranial MRI: bilateral periventricular leukomalacia, especially on the left, approximately 20 × 8 mm sagittal, and AP, the right is smaller, about 10 mm sagittal. Third ventricle and lateral ventricles of standard size and morphology.
- PATIENT 3: premature male patient, 47 months old, corrected age 45 months. He came for consultation at 33 months of age, corrected age to 31 months. Birth weight 1260 kg. Diagnosed with periventricular leukomalacia. Cranial MRI: periventricular leukomalacia, with increased frontal subarachnoid space. Third ventricle and lateral ventricles of normal size and morphology.

### 2.6. Intervention

**Figure 1:**
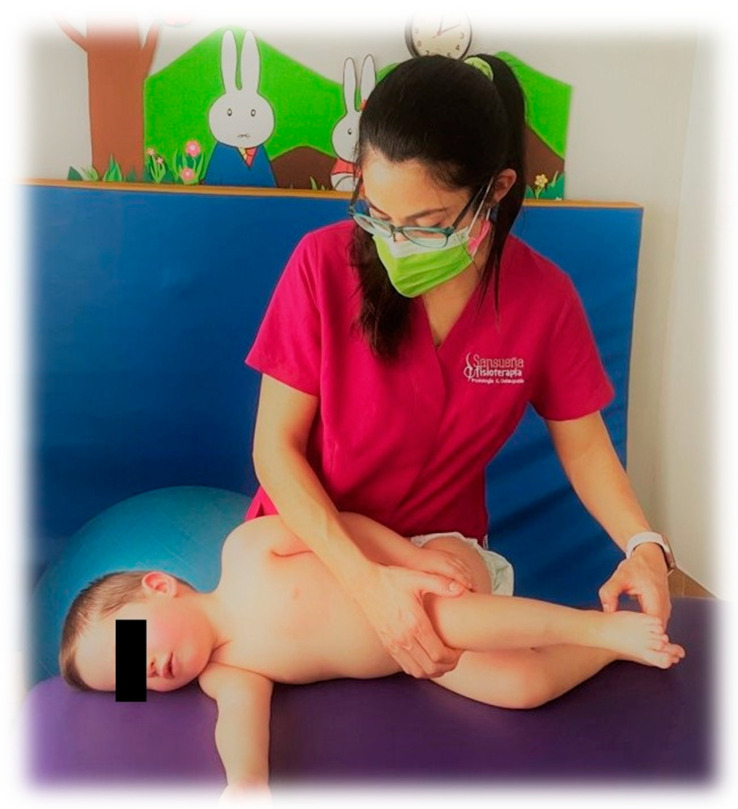
Second Reflex Flip Phase.

The intervention is based on the methods described by Vojta [6]. The first and second phases of the reflex tumbling, reflex reptation and first position of Vojta Therapy is performed with a triggering stimulus (Figure 1). Each work position is performed bilaterally for two minutes at a weekly frequency for eleven months. A detailed description of the intervention can be found in Supplementary File S1.

### 2.7. Evaluation

Each patient underwent the initial assessment at the start of treatment, during the initial physiotherapy assessment consultation, and the same assessment was repeated to obtain the results in November 2020, a summary can be check in Table 2.

The assessment tests that have been used are described below:

- Denver Developmental Screening Test II (DDST) [14]: The Denver Developmental Screening Test is one of the most widely used instruments to examine children’s developmental progress from birth to 6 years of age. It helps detect possible developmental problems, confirming suspected issues with objective measurement, and monitoring children at risk for developmental problems. Norms indicate when 25%, 50%, 75%, and 90% of children pass each task. Developmental assessment is based on the child’s performance and parent reports in four areas of functioning, fine motor, gross motor, personal-social, and language ability. The child’s exact age is calculated and marked on the assessment sheet, all those tasks that are intersected by the age line are assessed. The score is determined depending on whether the child’s response falls within or outside the expected normal range of success on each task for the age. The number of tasks on which the child falls below the expected range determines whether the child is classified as usual, suspect, or delayed.
- Bayley Scale of Infant Development (BSID) [15]: is a test that evaluates the child’s development in early childhood, from one month to three and a half years of age. With it, we can assess the cognitive, motor, and behavioral levels. Given the subject of the study, the assessment of the motor part has been carried out, this part of the BSID assesses the degree of body control, large muscle coordination, finer manipulatory skills of the hands and fingers, dynamic movement, postural imitation, and the ability to recognize objects by sense of touch. Specifically, items 19 to 50 have been taken as a reference to the capacities and ages of the patients. The aim is to detect motor development delays and act immediately to reduce damage to the central nervous system through cerebral neuroplasticity.

## 3. Results

**Figure 2:**
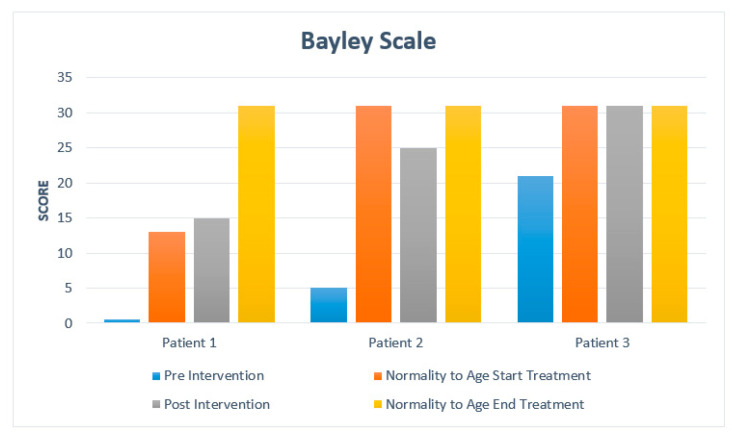
Patient score and comparison with normality based on Bayley Scale.

**Figure 3:**
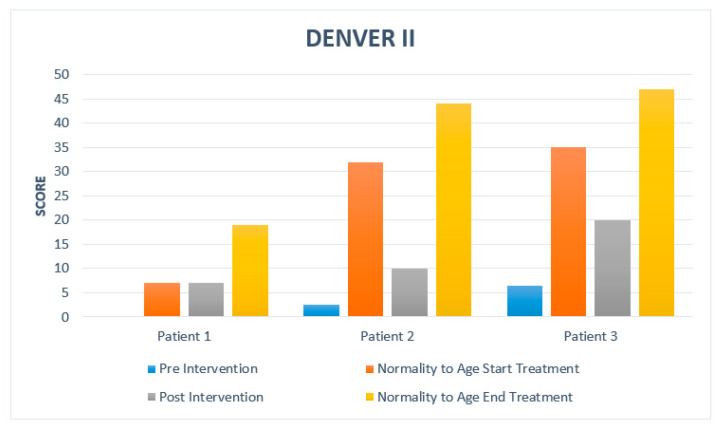
Patient score and comparison with normality based on DENVER Test II.

After the intervention, a clinically significant increase in the development of the patients is observed. Subsequently, the detailed progress of each of the patients studied is developed. A summary is presented in Table 3. Figure 2 and Figure 3 show a comparison concerning normality.

Patient 1: Bayley Scale: at 7 months, the age at which treatment was started, he obtained a score of 13, his assessment showed a score of 0; after treatment, the score got was 15. Denver test: the motor milestones considered normal for the age of 7 months would be: at a personal-social level: waving goodbye, banging two buckets in hands, grasping two buckets, passing bucket, raking. At a fine motor—adaptive level: looking for a string. At language level: specific daddy/mummy, spluttering, combined syllables. At large motor level: standing on something. At the beginning of the treatment, the Denver test was 0, so he did not perform milestones within the test. At the end of therapy, we obtained a Denver test of 7 months where the landmarks are: personal-social: waving goodbye, indicating wishes, playing pat-a-cake. In the fine-adaptive motor area: tapping two cubes in hands, finger shake. At language level: specific dada/mama, combined syllables. At large motor level: foot were leaning on something. 

Patient 2: Bayley Scale: with age at the start of treatment of 32 months, the score considered normal would be 31 points; in our patient, we had a score of 5, and after treatment, we obtained a score of 25. Denver test: with an age of 32 months, it is considered normal on a personal-social level: putting on a T-shirt, naming a friend. At the fine-adaptive motor level: tower of 8 cubes, imitating a vertical line. At language level: speak everything understandable, know four actions, name one color, use two objects, know two adjectives, know two steps, call four pictures. At large motor level: balance on unipodal support one second, long jump, balance on each foot two seconds. At the beginning of treatment, it was two and a half months: at the personal-social level: looking at hands. At the fine motor—adaptive level: hands together. At the language level: turning towards the rattle. At a large motor level: turning over, beginning to sit up, chest up, arm support, weighing on legs, sitting with head up, head raised 90°. After the treatment, the Denver test corresponds to 10 months, where at a personal—social level: drinking from a cup, playing with a ball. At the motor-adaptive level: puts bucket in cup, hits two buckets in hands. At language level: uses one word, says specific mama and dada. At large motor level: stands for 2 s.

Patient 3: Bayley Scale: with age at the start of treatment of 35 months, the score considered normal is 31 points; at the beginning, we obtained a score of 21, and after treatment, a normalized score of 31 points has been achieved. Denver test: at the age of 35 months, the following are considered normal at a personal-social level: brushing teeth without help, playing games, putting on a T-shirt, naming a friend. At fine-adaptive motor level: moving, tower of 8 cubes, imitating vertical line. At language level: understand four prepositions, talk about everything understandably, know four actions, use two and three objects, name one color, know two adjectives, know two steps, name four pictures. At the large motor level: balance on each foot for one second, long jump, unipodal balance for two seconds. At the beginning of the treatment, he presented a Denver test in 6 and a half months at a personal-social level: reaching for a toy and eating. At the fine motor and adaptive level: grasping two buckets, passing buckets, raking. At a language level: spluttering, combined syllables, non-specific mama/daddy, simple syllables. At gross motor level: standing with support. At the end of treatment, the Denver test is 20 months at personal—social level: washing/drying hands, brushing teeth with help, putting on clothes. At fine motor—adaptive level: tower of four and six cubes. Language level: medium-understandable speech, body parts, naming a picture, combining words, indicating two images. Large motor: throwing ball overhand, ball forward.

## 4. Discussion

Our study is based on three pediatrics patients affected with severe periventricular leukomalacia, suffering large motor deficits, which hinder adequate neurocognitive and motor development. Vojta Therapy was chosen as a method of intervention and promotion of neurodevelopment by the multidisciplinary team. The capacity of neuroplasticity at the level of the nervous system that patients of this age present and the pressure stimulation of the reflex locomotion patterns that with this therapy we can carry out changes in the neural networks in such a way that the locomotion patterns reflect are learned, influencing the brain stem and cerebellum, and motor patterns can be modulated [7,16].

One of the principles of Vojta Therapy is motor ontogenesis, which allowed the discovery of reflex locomotion [17]. After the intervention, positive changes have been generated in the neuromotor development of all the cases studied, the stimulation of the patterns described by Vojta stimulated the automatic control of posture, the activation of straightening and mobility mechanisms [17] generating essential changes in the gross motor development of the patients studied. We have been able to verify these changes with both scales of development, where gross motor skills have undergone a significant increase, the patients did not meet the motor milestones appropriate for their age and in two of the cases there was hardly any movement. During the treatment, all patients have acquired essential motor milestones for their neurodevelopment such as crawling, rolling over, sitting held with reach of objects, with respect to walking, patient three has started walking without help and patient two has started walking with support. The acquisition of these motor milestones can be considered of great relevance, being clinically significant [18,19]. Despite the fact that they are not yet in their developmental age, the clinical evolution is favorable and essential for the patients since with this they begin to develop the first motor, personal—social and language milestones that will allow them to advance in their neurodevelopment [18,19].

We have been able to verify this with both scales of development, although not all cases have reached the motor age that they should acquire for their period. Still, the evolution is favorable and should be analyzed in the future until the limit is verified concerning normality.

Other similar interventions through Vojta Therapy have also generated positive effects on the neurological disorders of the child [13,20,21]. Specifically focused on improving posture [21], gait [20], and respiratory capacity [13]. However, there is a scarcity of research articles on Vojta Therapy, even though it is a prevalent therapy in clinical practice, clinicians must influence studies that raise the level of evidence of this therapy, the significant heterogeneity of patients with neurological problems, and the necessary treatment individualization makes it difficult to carry out quality studies with large samples, neurological pathologies are broad spectrum of study, where it is unlikely to find large samples with pathologies that have the same symptoms, signs and deficits. In this sense, our article has limited evidence since it consists of a series of cases that do not have enough power to be extrapolated. Further, we believe it is important that future studies assess the impact of progress in neuromotor development on positive changes in the quality of life and psychological state of caregivers/parents, this being an aspect that is sometimes forgotten, but of vital importance for stimulation at home. and communication with the therapeutic team.

After analyze the results, we might suggest that Vojta therapy applied in patients who have been diagnosed with periventricular leukomalacia could be included as a treatment option for their neurodevelopment, since we have verified through the measurement of the development scales that there are advances in the acquisition of motor milestones that positively influence the performance of different vital activities of daily life.

## 5. Conclusions

Vojta Therapy in patients with periventricular leukomalacia with delayed neuromotor development seems to generate adequate progression in the motor area, acquiring motor milestones such as crawling, rolling over, and sustained sitting with object reach. Other personal, social and language milestones could be stimulated as a consequence of the clinical benefit achieved.

## Figures and Tables

**Table 1:** Characteristics of study patients.

No. Patient	Medical Diagnosis	Date of Birth	Age Corrected End of Treatment	Age Corrected Start of Treatment	Birth Weight	Sex
1	Leukomalacia	20 January 2019	19 months	7 months	1398 Kg	Male
2	Leukomalacia	8 October 2016	44 months	32 months	1750 Kg	Male
3	Leukomalacia	26 December 2016	47 months	35 months	1260 Kg	Male

**Table 2:** Evaluation and sessions.

No. Patient	1st Evaluation	Last Evaluation	Number of Sessions
1	18 November 2019	11 November 2020	53
2	25 November 2019	12 November 2020	52
3	30 October 2019	19 November 2020	59

**Table 3:** Summary of intervention and results.

No. Patient	Age Start Treatment	Age End of Treatment	DENVER II Pre	DENVER II Post	Bayley Scale Pre	Bayley Scale Post
1	7	19	0 months	7 months	0	15
2	32	44	2.5 months	10 months	4	25
3	35	47	6.5 months	20 months	21	31

## Data Availability

De-identified individual participant data generated during this study are available from the investigators on reasonable request with the publication. Requests should be directed to the corresponding author by email.

## Supplementary Materials

The following are available online at https://www.mdpi.com/article/10.3390/medicina57111149/s1, File S1: Vojta Therapy Intervention.
