# Supplementary material for: Vojta Therapy in Neuromotor Development of Pediatrics Patients with Periventricular Leukomalacia: Case Series
Source: Medicina (Kaunas). 2021 Oct 23;57(11):1149. doi: 10.3390/medicina57111149 (PMC8624747; doi:10.3390/medicina57111149)
Supplement: Supplementary file 1 [file medicina-57-01149-s001.zip › medicina-1398510-supplementary.pdf]

VOJTA THERAPY INTERVENTION

- **<u>1st Phase of the reflex flip</u>:** starting position: dorsal decubitus, head rotated to one side, facial arm in slight extension and adduction: after the triggering stimulus, abduction and external rotation with elbow extension and supination of the forearm, an act that will be resisted by the therapist, for which the muscles involved will be: pectoralis major, anterior deltoid, serratus anterior and subscapularis. The supraspinatus and latissimus dorsi will also contract slightly, preventing the arm from falling on the thorax. Nuchal arm: starting position: slight abduction, contraction of the muscles: posterior deltoid, triceps brachii, and teres major, causing the support to move to the nuchal shoulder to carry out the 2nd phase of the rollover and having as a response the abduction of the arm to 90º, flexion of the elbow to 90º and unfolding of the hand. Nuchal and facial leg: in relaxed extension, after stimulation, perform flexion with slight abduction and dorsal extension of the feet in the midline. Facial leg: after stimulation: will remain in slight abduction and flexion, maintained by the contraction of the adductors and in balance with the abductors and external rotators of the thigh. Nuchal leg: after stimulation, extension and external rotation of the leg occurs, stabilization of the hip with a simultaneous extension of the leg due to contraction of the muscles: gluteus medius, tensor fascia lata, quadriceps femoris, and adductors. The primary triggering stimulus is the pectoral or mammillary point: pressure is applied in the direction of the spine, on the thorax in the intercostal area, on the mammillary line at the level of the insertion of the diaphragm on the facial side. After stimulation: the head is rotated towards the contralateral side, which is resisted by the therapist, simultaneously causing a symmetrical and transitory extension of the neck, which is caused by the contraction of

the following musculature: sternocleidomastoid on the facial side, longissimus on the neck.

Another triggering stimulus used was the mandibular point: at the level of the posterior edge of the mylohyoid in a ventral direction, causing the tongue to deviate towards the nuchal side and triggering the act of swallowing together with an activation of the ventral musculature. Finally, we have also stimulated the acromial point on the nuchal side, thereby triggering activation of the scapular area together with the reaction of abduction of the arm to 90º, flexion of the elbow to 90º degrees, and deployment of the hand on the nuchal side.

All this causes an activation of the abdominal wall, rectus abdominis, transversus abdominis and oblique abdominis muscles.

2nd phase of the reflex roll: starting position: lateral decubitus, support on the lower shoulder and the extended leg after the 1st phase of the reflex roll. Facial arm (upper): after the triggering stimulation on the inner edge of the scapula towards the spine and anterior triggers an oblique arm movement towards the head: abduction and external rotation of the arm, supination of the elbow, and extension with radial deviation of the hand, this movement is resisted by the therapist, thereby causing an isometric contraction of the musculature of the scapular area and the trunk. Nuchal arm (lower): starting position: perpendicular to the axis of the body, it exerts pressure on the medial epicondyle of the humerus, facilitating, together with the rest of the stimuli, the straightening of the shoulder girdle, pronation of the arm, extension, radial deviation and separation of the fingers of the nuchal arm. Facial leg (upper): stimulation is performed on the medial femoral epicondyle in the direction of the upper hip, and together with stimulation of the inner edge of the scapula, the leg adopts flexion and abduction and the foot dorsal flexion. Nuchal (lower) leg: it will extend in external rotation and abduction, thus stimulating the

external femoral condyle and provoking straightening from lateral decubitus, the gluteus medius muscle, and the external hip rotators will undergo elongation, the foot will adopt dorsal flexion and supination with flexion of the toes. In addition to the stimulation points described above, we add pressure on the anterior superior iliac spine, activating the entire coordinator complex due to spatial summation.

Reflex reaction: starting position: ventral decubitus, head rotated, which we will call the facial side and the contralateral side the nuchal side. Facial arm: in flexion, with support on the medial epicondyle, which produces contraction of the following muscles: scapular area: lower trapezius, serratus anterior and rhomboid, producing fixation of the scapula. Shoulder joint, contraction of posterior deltoid, triceps brachii, teres major, and latissimus dorsi muscle in its transverse part, causing the reflex action of the arm backward, the biceps brachii and the acromial part of the deltoid secure the shoulder joint. The therapist applies resistance causing a change in muscle action, the musculature performs an isometric contraction, and the elbow becomes a fixed point, changing the muscle contraction towards the new set point, causing contraction of the anconeus, biceps brachii, and brachialis anterior. Straightening of the trunk and anti-gravity action: pectoralis major, coracobrachialis, and subscapularis by the contraction of the muscles. The supraspinatus and infraspinatus muscles are used to balance the internal and external rotation of the shoulder joint. The nuchal arm is extended in pronation and internal rotation, thus distending the infraspinatus, anterior deltoid, and supinator muscles; a forward movement will be produced, given by the contraction of the following musculature: scapular area, upper trapezius, upper trapezius, deltoid, and supinator muscles: upper trapezius, anterior deltoid, serratus anterior, in the shoulder: anterior deltoid, pectoralis minor, and infraspinatus, in the elbow: anterior brachialis, biceps brachii, brachioradialis, and supinator, at the level of the wrist and fingers: the

deployment of the hand will occur. Facial leg: is in semi-flexion and external rotation of the hip, the medial condyle will be the fixed point thereby causing the straightening of the pelvis, the muscles involved are: in the pelvic girdle: iliac psoas, rectus femoris, and sartorius, synergists will have the thigh abductors and external and internal hip rotators, preventing the position in anterior torsion of the neck of the femur the gluteus medius. This is followed by a change of muscular action, which, together with the straightening of the pelvis, produces dorsal flexion of the ankle. Nuchal leg: starting position: semi-flexion and external rotation, the extension will be made by the pressure on the back and top of the heel against the base of support, the main muscles that act are: gluteus medius and tensor fascia lata and adductors as synergists in the extension of the leg. The gluteus maximus, triceps suralis, and tibialis posterior are also contracted, which causes supination and flexion of the toes.

All this is carried out by stimulating the points described by Vojta, known as triggering stimuli, which in our intervention were: calcaneal point nuchal leg: applying pressure in the direction of the nuchal knee and epicondyle facial arm: applying pressure in the direction of the facial shoulder, thus activating the entire coordination complex in the neonatal period.

- 1st position: starting position: buttocks position on heels, with trunk flexion and feet outside the edge of the stretcher, rotation head. Nuchal and facial arm similar to that explained in the reflex reptation. Nuchal and facial leg: buttocks on heels. Triggering stimuli: calcaneal point on nuchal leg medially and cranially and epicondyle on the facial arm, medially towards the facial shoulder. Activation of the trunk erector muscles, the musculature of the shoulder girdle and hamstring muscles, increased elbow and knee support.
